# Regional and time course differences in sweat cortisol, glucose, and select cytokine concentrations during exercise

**DOI:** 10.1007/s00421-023-05187-3

**Published:** 2023-04-02

**Authors:** Michelle A. King, Shyretha D. Brown, Kelly A. Barnes, Peter John D. De Chavez, Lindsay B. Baker

**Affiliations:** 1 Gatorade Sports Science Institute, PepsiCo R&D Life Sciences, Barrington, IL USA; 2 Data Science and Analytics, PepsiCo R&D, Barrington, IL USA; 3 Gatorade Sports Science Institute, PepsiCo R&D Life Sciences, Valhalla, NY USA

**Keywords:** Excretion rate, Biomarkers, Thermoregulation, Chemokines

## Abstract

**Introduction:**

The use of sweat as a biofluid for non-invasive sampling and diagnostics is a popular area of research. However, concentrations of cortisol, glucose, and cytokines have not been described across anatomical regions or as time progresses throughout exercise.

**Purpose:**

To determine regional and time course differences in sweat cortisol, glucose, and select cytokines (EGF, IFN-γ, IL-1β, IL-1α, IL-1ra, TNF-α, IL-6, IL-8, and IL-10).

**Methods:**

Sweat was collected with absorbent patches from eight subjects (24–44 y; 80.2 ± 10.2 kg) on the forehead (FH), right dorsal forearm (RDF), right scapula (RS), and right triceps (RT) at 0–25 min, 30–55 min, and 60–85 min during 90 min of cycling (~ 82% HR_max_) in a heated chamber (32 °C, 50% rh). ANOVA was used to determine the effect of site and time on outcomes. Data are reported as LS means ± SE.

**Results:**

There was a significant effect of location on sweat analyte concentrations with FH having higher values than most other regions for cortisol (FH: 1.15 ± 0.08 ng/mL > RDF: 0.62 ± 0.09 ng/mL and RT: 0.65 ± 0.12 ng/mL, *P* = 0.02), IL-1ra (*P* < 0.0001), and IL-8 (*P* < 0.0001), but lower concentrations for glucose (*P* = 0.01), IL-1α (*P* < 0.0001), and IL-10 (*P* = 0.02). Sweat IL-1β concentration was higher on the RS than RT (*P* < 0.0001). Sweat cortisol concentration increased (25 min: 0.34 ± 0.10 ng/mL < 55 min: 0.89 ± 0.07 ng/mL < 85 min: 1.27 ± 0.07 ng/mL; *P* < 0.0001), while EGF (*P* < 0.0001), IL-1ra (*P* < 0.0001), and IL-6 (*P* = 0.02) concentrations decreased over time.

**Conclusion:**

Sweat analyte concentrations varied with time of sampling and anatomical region, which is essential information to consider when conducting future work in this area.

**Clinical trial identifier:**

NCT04240951 registered January 27, 2020.

**Supplementary Information:**

The online version contains supplementary material available at 10.1007/s00421-023-05187-3.

## Introduction

There is considerable interest in the use of sweat as a biofluid for non-invasive sampling and continuous diagnostics to track patient or athlete status. Sweat content has the potential to deliver insights about underlying physiological and metabolic processes that reflect changes in exercise performance and health (Baker 2019). Specifically, the presence of cortisol, glucose, and specific signaling proteins (e.g., cytokines) in sweat are particularly attractive as biomarkers. Cortisol and glucose are biomarkers of particular interest due to their relationship to the stress response and overall health. Sweat cortisol monitoring may be advantageous in the prevention of disease or acceleration of recovery, especially because its prolonged elevation in the circulation can promote a variety of adverse conditions (Sternberg 2001). Sweat glucose could provide a practical and non-invasive alternative to blood glucose monitoring. Cytokines and chemokines in sweat have also gained attention because of their potential to serve as markers of immune function (Cizza et al. 2008; Didierjean et al. 1990; Katchman et al. 2018; Marques-Deak et al. 2006) or even indicators of local skin inflammation (Aranyosi et al. 2021; Dai et al. 2013).

Although there is great interest in the ability of sweat biomarkers to provide meaningful user feedback, little is known about the concentration and excretion rate of sweat components, with the exception of sodium and other electrolytes (Baker and Wolfe 2020). To elucidate the relevance and future utility of specific biomarkers in sweat, regional differences in concentration, time course changes, and excretion rates must first be described. Different regions of the body are known to vary drastically in both local sweat mineral concentrations and local sweating rate (LSR) (Patterson et al. 2000; Sato and Dobson 1970). For example, it is clear that certain minerals, such as sodium, calcium, chloride, magnesium, iron, copper, and zinc, are found in different concentrations across the body (Aruoma et al. 1988; Patterson et al. 2000; Sato and Dobson 1970). Likewise, there is considerable regional variability in LSR, with the forehead (FH) producing the highest reported values (Sato and Dobson 1970). For these reasons, sweat mineral concentrations and excretion rates from different regions should not be used interchangeably, nor should they be generalized to the whole body. However, regional variability in sweat cortisol, glucose, and cytokine concentrations has not been studied.

Timing of sample collection is another important methodological consideration. Several studies have found higher concentrations of certain sweat analytes in initial versus subsequent sweat collections (Boysen et al. 1984; Brune et al. 1986; Ely et al. 2011; Paulev et al. 1983). At the onset of sweating, trace minerals (Brune et al. 1986; Ely et al. 2011; Montain et al. 2007), metabolites (Komives et al. 1966), cytokines (Sato and Sato 1994), and cortisol (Terao and Katayama 2016) may be artificially elevated, because residual contents of the sweat duct, sebum secretions, epidermal cells, and/or other skin surface contaminants may be included in the initial sample (Baker 2019). Depending on the timing of sample collection, this may lead to an overestimation of the concentration in the initial sweat sample compared with subsequent samples (Ely et al. 2011; Freyberg and Grant 1937; Robinson and Robinson 1954). However, inconsistencies in sweat sample location, timing, and methods of collection and analysis prevent comparisons across studies.

Therefore, the objective of this study was to determine the influence of anatomical region and timing of sample collection on sweat analyte concentrations and excretion rates. To examine this, we measured sweat cortisol, glucose, and select cytokine concentrations (EGF, IFN-γ, IL-1β, IL-1α, IL-1ra, TNF-α, IL-6, IL-8, and IL-10) from four different regions over three different time points during 90 min of exercise in the heat. We hypothesized that as the duration of exercise progressed, select cytokines would demonstrate a flushing of the sweat gland with the highest concentrations early in exercise and the lower concentrations later in exercise. Cortisol concentrations may follow a pattern similar to cytokines (Steensberg et al. 2003; Terao and Katayama 2016), although exertional heat stress may impact this time course (Nehlsen-Cannarella et al. 1997). We anticipated glucose concentrations to remain relatively stable over time (Boysen et al. 1984). Further, we hypothesized that the FH would display the highest LSR and excretion rate for all analytes (Sato and Dobson 1970).

## Methods

### Subjects

Eight moderately trained subjects (7 males, 1 female) participated in this study. This study (clinical trial identifier: NCT04240951) was approved by the Sterling Institutional Review Board (Atlanta, GA, sterlingirb.com) and has therefore been performed in accordance with the ethical standards in the Declaration of Helsinki. Before providing written informed consent, subjects were informed of the experimental procedures and associated risks. Trials were completed in the winter (January–March) in northeast Illinois to minimize seasonal acclimatization effects.

### Preliminary screening

Upon the first visit to the laboratory (~ 0700–0800 h), subjects participated in a medical screening that consisted of measurements of nude body mass, height, resting heart rate, resting blood pressure, and ~ 8 h fasted blood glucose concentration. Subjects also completed a graded exercise test to assess cardiovascular health (12-lead ECG, Schiller AT-10 Plus; Schiller America, Doral, FL), maximum heart rate (HR_max_), and maximum aerobic capacity ($V\dot{O}_{2\max }$) (MOXUS; AEI Technologies, Pittsburgh, PA) on a cycle ergometer (Velotron SRAM, Pro, Chicago, IL).

### Experimental procedures

Subjects reported to the laboratory at 0900 or 1300 h after abstaining from caffeine, alcohol, and vigorous exercise for 24 h. Subjects were asked to fast from food, but drink 16 oz of water 2 h before the experimental trial to promote a well-hydrated state. Upon reporting to the laboratory, a urine sample was collected to assess baseline urine-specific gravity (USG; Atago Pen Refractometer, 3741‐E03 Saitama, Japan). Next, subjects’ skin on the forehead (FH), right dorsal forearm (RDF), right scapula (RS), and right triceps (RT) was shaved (if necessary) and cleaned with alcohol to prepare for later sweat patch application. These locations were chosen, because they are among those most commonly reported in the literature (Baker and Wolfe 2020) and the RDF and RT in particular are likely regions for wearable devices. Nude body mass was measured using a digital platform scale (KCC300 platform and IND439 reader; Mettler Toledo, Columbus, OH) to the nearest 0.01 kg. During the trial, subjects cycled on an ergometer (Velotron SRAM, Pro, Chicago, IL) for 90 min in a heated chamber (32 °C, 50% rh) at 82 ± 6% HR_max_. Subjects were allowed to gradually warm up to their target heart rate (HR) and power output in the first ~ 10–20 min. HR was monitored using telemetry (Polar Electro RS400; Lake Success, NY) every 10 min along with ratings of perceived exertion (RPE), power output (Watts), and cadence (revolutions per min). Subjects were allowed to drink a sports drink (6% carbohydrate solution) ad libitum throughout the duration of exercise. After exercise and removal of all sweat patches, subjects were asked to towel dry before obtaining a final body mass measurement.

### Sweat collection

**Fig. 1:**
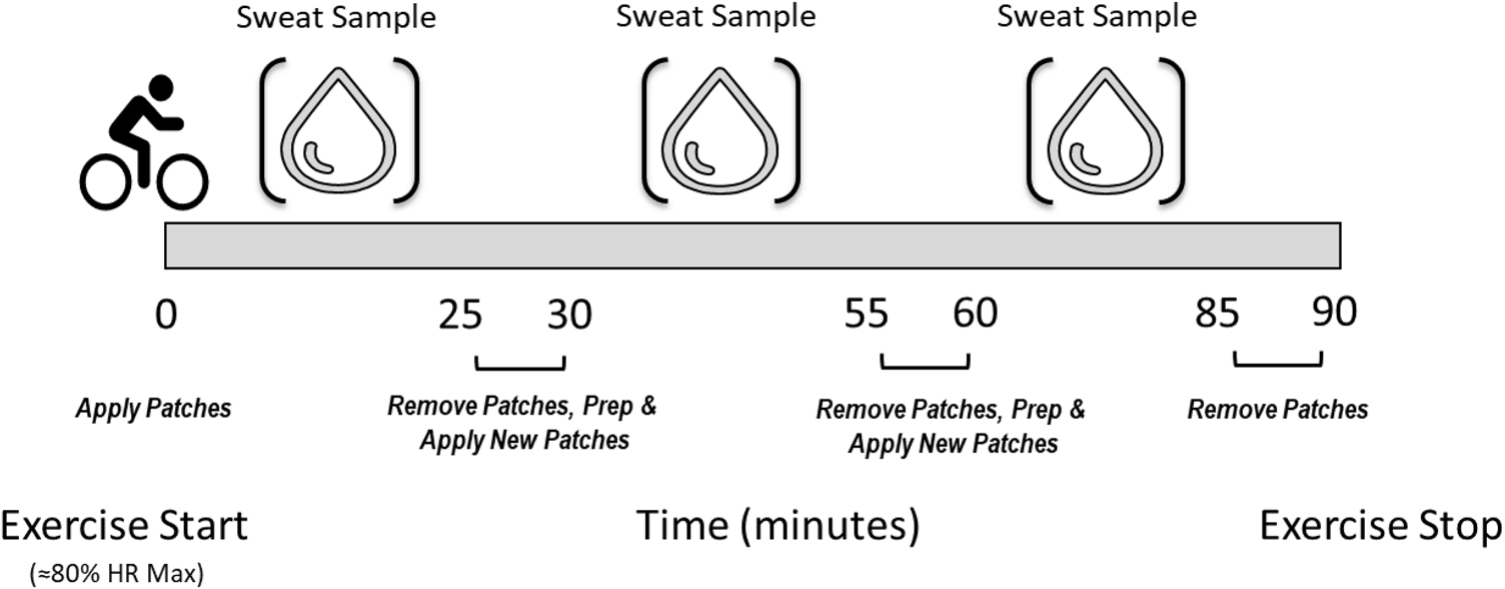
Protocol schematic showing time course of sweat patch application and removal

The standard absorbent patch method (11.9 cm^2^ absorbent pad, 3 M Tegaderm™ + Pad) (Baker et al. 2018) was used to collect regional sweat for this study. Anatomical location of patches were as follows: RDF was placed on the posterior midline of the forearm approximately half way between the styloid process of the radius and olecranon process; RT was placed on the posterior midline of the upper arm, approximately half way between the acromion and the olecranon process; RS was placed directly below the scapular spine and lateral to the medial border of the scapular spine; FH was placed in the midline of the frontal bone above the glabella but below the hairline. In the heated chamber immediately before exercise, FH, RDF, RS, and RT skin regions were cleaned with deionized water and wiped dry. Deionized water temperature equilibrated approximately to the temperature of the environmental chamber (32 °C) before it was used to clean the skin. Absorbent patches were applied and removed from each site according to the schedule shown in Fig. 1. Samples were collected during 0–25 min (25), 30–55 min (55), and 60–85 min (85) of the 90 min cycling protocol. Once the patch was removed, the skin was left uncovered until the next patch application time. Before the next application, the skin was wiped with deionized water on electrolyte-free gauze and immediately wiped dry before the patch was applied. When necessary, an elastic net dressing (Surgilast; Derma Sciences, Princeton, NJ) was put on the RDF to ensure that the patch remained adhered to the skin. Once patches were removed, the absorbent pad was instantly detached from the Tegaderm with clean forceps and placed in an air-tight plastic tube (Sarstedt Salivette, Nümbrecht, Germany). LSR (mg/cm^2^/min) was measured gravimetrically using Mettler Toledo Balance XS204 (Columbus, OH). Sweat was extracted from the absorbent patch via centrifuge (Eppendorf, Centrifuge 5810 R, Germany) (1000 relative centrifugal force, 10 min, 17 °C). Aliquots of sweat were frozen at − 80 °C until analyses.

### Sweat sample handling

A total of 96 sweat samples (8 subjects × 4 sites × 3 time points) were collected. However, because of low sample volumes for some subjects (especially from the RT), fewer than 96 samples were analyzed for each analyte (IL-6, *n* = 92; EGF, *n* = 91; IL-1ra, *n* = 90; IL-1α, *n* = 90; IL-1ꞵ, *n* = 88; IL-8, *n* = 85; cortisol, *n* = 81; IL-10, *n* = 76, glucose, *n* = 56). The final sample sizes for each anatomical location and time point are shown in Figures S1-10. Once ready for analysis, sweat samples were removed from the freezer and allowed to thaw at room temperature. After thawing, sweat samples were vortexed and/or centrifuged depending on the requirements of the chosen analytical method.

### ELISA assays

ELISA was used for the detection of cortisol (Invitrogen, ThermoFisher Scientific) and glucose (Caymen Chemical Co.) concentrations. All ELISA assays were performed according to the manufacturer’s instructions. For cortisol, 12.5 μL of sweat sample (1:4 dilution), in duplicate, was assayed. Assay buffer (for detecting non-specific binding) cortisol conjugate, and cortisol antibody were added to the samples in each well and incubated for 1 h at room temperature. After manual washing of wells 4 times, tetramethylbenzidine (TMB) substrate was added and incubated for 30 min which produced a colorimetric signal. Stop solution was added for 10 min and the absorbance was read at 450 nm using a plate reader. Intra-assay coefficient of variation was 1.2%. For glucose, 15 μL of sweat sample, in duplicate, was added to assay buffer in each well. The reaction was initiated by adding enzyme mixture, and the plate was covered and incubated for 10 min at 37 °C. Absorbance was read at 520 nm using a plate reader. Intra-assay coefficient of variation was 2.0%. Both assays were read using Bioteck Cytation 3 Multi-Reader (Bioteck, Winooski, VT) with Gen5 software.

### Multiplex assay

Epidermal growth factor (EGF), interferon (IFN)-γ, interleukin (IL)-1β, IL-1α, IL-1 receptor agonist (ra), tumor necrosis factor (TNF)-α, IL-6, IL-8, and IL-10 were measured using magnetic bead-based immunoassay Multiplex. The Multiplex assay was performed according to the manufacturer’s instructions (Milli-Q, Millipore Sigma, Burlington, MA). Briefly, 25 μL of sweat sample, in duplicate, was assayed. The Multiplex assay involved beads of defined spectral properties conjugated to protein-specific capture antibodies and added with the samples (including standards of known protein concentration and control and test samples) into the wells of a microplate. The target proteins bound to the capture antibodies for an overnight incubation. After manual washing of the beads using a magnetic plate, protein-specific biotinylated detector antibodies were added and incubated with the beads for 1 h. Then, excess biotinylated detector antibodies were removed, and a streptavidin-conjugated fluorescent protein, R-phycoerythrin (SAV-RPE), was added and incubated for 30 min. SAV-RPE bound to the biotinylated detector antibodies, forming a four-member, solid-phase sandwich. After washing to remove unbound SAV-RPE, the beads were analyzed, and standard curves (EGF = 0.81% CV; IFN-γ = 0.83% CV; IL-1β = 0.41% CV; IL-1α = 0.23% CV; IL-1ra = 0.17% CV; TNF-α = 0.16% CV; IL-6 = 0.21% CV; IL-8 = 0.02% CV; IL-10 = 0.62% CV) along with individual well concentrations were determined using the Magpix multiplex system with xPonent software (Luminex, Austin, TX).

### Calculations

LSR was calculated from sweat mass over patch surface area (11.9 cm^2^) and exercise duration. Excretion rates were calculated as the product of analyte concentration and LSR. Whole-body sweating rate was calculated from the change in pre- to post-exercise nude body mass, corrected for fluid intake (difference in drink bottle mass from pre- to post-exercise to the nearest 0.01 kg using a digital scale; model PG802, Mettler Toledo, Columbus OH) estimated respiratory water loss, and estimated metabolic mass loss (Cheuvront and Kenefick 2017).

### Statistical analysis

Statistical analyses were performed using Minitab 19 Statistical Software (Minitab, Inc., State College, PA). Shapiro–Wilk tests were used to assess the normality of the residuals. The Box–Cox transformation technique was used to identify the optimal data transformation where needed. Analysis of variance (ANOVA) was used to determine site, time, and site*time interaction effects on LSR, sweat analyte concentrations, and sweat analyte excretion rates. ANOVA was followed by Tukey’s HSD post hoc comparisons, where main effects were found. The significance level was set at *P* < 0.05. Data are presented as Least Squares (LS) mean ± SE. Due to the exploratory nature of this study, a post hoc analysis was performed for cortisol (primary analyte of interest), which determined an effect size of 0.29.

## Results

### Subject characteristics

Participants’ (*n* = 8) age, body mass, height, body surface area, HR_max_, $\dot{V}O_{2\max }$_,_ USG, and body mass lost were 34 ± 7 yr, 80.15 ± 10.18 kg, 181.4 ± 4.7 cm, 2.0 ± 0.12 m^2^, 177 ± 15 bpm, 52.2 ± 6.4 ml/kg/min, 1.012 ± 0.008, and 1.31 ± 0.43%, respectively. During 90 min of exercise, subjects cycled at an average of 82 ± 6% HR_max_ with an RPE of 13 ± 1. Although there was a brief warm up phase of ~ 10–20 min, the average %HR_max_ and power output did not change over the duration of exercise (*P* = 0.10 and *P* = 0.78, respectively).

### Sweating rate

Overall, both local and whole-body sweating rate were congruent with previously published normative data in a similar population (Baker et al. 2016, 2018). Whole-body sweating rate was 1.06 ± 0.02 L/h (0.85 ± 0.09 mg/cm^2^/min), and as expected, the FH produced the highest LSR and the RT the lowest (*P* < 0.0001) (Table 1). The effect of time on LSR was significant, where 25 was lower than the 55 and 85 min time points (*P* ≤ 0.0001). Generally, LSR increased from the 25 to the 55 min time point but then reached a plateau or stabilized.

**Table 1:** Local sweating rate

	Site			
	Forehead	Right dorsal forearm	Right scapula	Right triceps
LSR* (mg/cm^2^/min)	3.64 ± 0.14^a^	1.75 ± 0.14^b^	1.55 ± 0.14^b^	0.88 ± 0.14^c^

	Time		
	25 min	55 min	85 min
LSR* (mg/cm^2^/min)	1.48 ± 0.12^a^	2.25 ± 0.12^b^	2.14 ± 0.12^b^

Values are LS means ± SE. ANOVA followed by Tukey’s HSD post hoc comparisons, where appropriate

*Significant main effects of site and time (*p* < 0.05)

^a,b,c ^LS means for sites or time points sharing the same letter are not significantly different

### Sweat cortisol

Cortisol concentrations were affected by location (*P* < 0.05) (Table 2). FH concentration was significantly higher than both the RDF and the RT, but not different from the RS. There was also an effect of time on cortisol concentration (*P* < 0.0001) (Table 3). Cortisol concentrations at both 25 and 55 were significantly lower than 85, and the concentration at 25 was lower than 55. There was an effect of location on cortisol excretion rates (*P* < 0.0001) (Table 4), with FH significantly higher than all other sites. In addition, RT had a significantly lower excretion rate than RDF and RS. There was an effect of time on cortisol excretion rates (*P* < 0.0001) (Table 5). Similar to cortisol concentration patterns, cortisol excretion rates at 25 were significantly lower than 55 and 85. Cortisol excretion rates were also significantly lower at 55 compared to 85. There were no significant site*time interaction effects for cortisol (*P* > 0.05).

**Table 2:** Sweat analyte concentrations across sites

	Forehead	Right dorsal forearm	Right scapula	Right triceps
Cortisol (ng/mL)^†^*	1.15 ± 0.08^a^ (3.15)	0.62 ± 0.09^b^ (1.85)	0.91 ± 0.08^ab^ (2.48)	0.65 ± 0.12^b^ (1.92)
Glucose (mg/dL)*	0.37 ± 0.01^a^	0.41 ± 0.01^b^	0.41 ± 0.01^b^	0.45 ± 0.01^b^
EGF (pg/mL)^†^	3.71 ± 0.10^a^ (41.0)	3.77 ± 0.11^a^ (43.5)	3.92 ± 0.10^a^ (50.3)	3.94 ± 0.11^a^ (51.5)
IL-1α (pg/mL)^†^*	6.85 ± 0.13^a^ (940)	8.40 ± 0.14^b^ (4463)	8.82 ± 0.13^b^ (6739)	8.63 ± 0.15^b^ (5577)
IL-1β (pg/mL)^†^*	− 0.69 ± 0.02^ab^ (4.72)	− 0.74 ± 0.02^ab^ (3.51)	− 0.67 ± 0.02^a^ (5.14)	− 0.77 ± 0.03^b^ (3.00)
IL-1ra (pg/mL)*	1017 ± 83^a^	303 ± 88^b^	288 ± 83^b^	483 ± 93^b^
IL-6 (pg/mL)	0.105 ± 0.004^a^	0.129 ± 0.004^a^	0.127 ± 0.004^a^	0.132 ± 0.004^a^
IL-8 (pg/mL)^†^*	1.39 ± 0.23^a^ (4.01)	− 2.23 ± 0.24^b^ (0.11)	− 1.60 ± 0.23^b^ (0.20)	− 2.14 ± 0.29^b^ (0.12)
IL-10 (pg/mL)^†^*	0.48 ± 0.01^a^ (0.23)	0.54 ± 0.01^b^ (0.29)	0.52 ± 0.01^b^ (0.28)	0.50 ± 0.01^ab^ (0.25)

Values are LS means ± SE

^†^Data were analyzed on a transformed scale and LS means are provided on the original scale in parenthesis. Box–Cox transformation technique was used to identify the optimal data transformation, where needed. ANOVA followed by Tukey’s HSD post hoc comparisons, where appropriate

*Significant main effects of site (*p* < 0.05)

^a,b,c^ LS means for sites sharing the same letter are not significantly different

**Table 3:** Sweat analyte concentrations across time

	25 min	55 min	85 min
Cortisol (ng/mL)^†^*	0.34 ± 0.10^a^ (1.40)	0.89 ± 0.07^b^ (2.42)	1.27 ± 0.07^c^ (3.57)
Glucose (mg/dL)	0.42 ± 0.01^a^	0.40 ± 0.01^a^	0.41 ± 0.01^a^
EGF (pg/mL)^†^*	4.83 ± 0.09^a^ (125.4)	3.69 ± 0.09^b^ (40.0)	2.99 ± 0.09^c^ (19.9)
IL-1α (pg/mL)^†^	8.33 ± 0.12^a^ (4155)	8.24 ± 0.12^a^ (3777)	7.95 ± 0.12^a^ (2836)
IL-1β (pg/mL)^†^	− 0.71 ± 0.02^a^ (4.05)	− 0.69 ± 0.02^a^ (4.56)	− 0.74 ± 0.02^a^ (3.42)
IL-1ra (pg/mL)*	659 ± 76^a^	515 ± 75^ab^	395 ± 73^b^
IL-6 (pg/mL)*	0.131 ± 0.004^a^	0.121 ± 0.003^ab^	0.118 ± 0.003^b^
IL-8 (pg/mL)^†^	− 1.38 ± 0.22^a^ (0.25)	− 0.88 ± 0.21^a^ (0.41)	− 1.17 ± 0.21^a^ (0.31)
IL-10 (pg/mL)^†^	0.53 ± 0.01^a^ (0.28)	0.51 ± 0.01^a^ (0.26)	0.50 ± 0.01^a^ (0.25)

Values are LS means ± SE

^†^Data were analyzed on a transformed scale and LS means are provided on the original scale in parenthesis. Box–Cox transformation technique was used to identify the optimal data transformation, where needed. ANOVA followed by Tukey’s HSD post hoc comparisons, where appropriate

*Significant main effects of time (*p* < 0.05)

^a,b,c ^LS means for time points sharing the same letter are not significantly different

**Table 4:** Sweat analyte excretion rates across sites

	Forehead	Right dorsal forearm	Right scapula	Right triceps
Cortisol (pg/cm^2^/min)^†^*	2.36 ± 0.11^a^ (10.63)	1.15 ± 0.12^b^ (3.15)	1.30 ± 0.11^b^ (3.66)	0.51 ± 0.17^c^ (1.66)
Glucose (ng/cm^2^/min)*	14.46 ± 1.16^a^	6.28 ± 1.18^b^	5.59 ± 1.23^b^	6.94 ± 2.03^b^
EGF (pg/cm^2^/min)^†^*	− 2.03 ± 0.11^a^ (0.13)	− 2.66 ± 0.12^b^ (0.07)	− 2.61 ± 0.12^b^ (0.07)	− 3.27 ± 0.13^c^ (0.04)
IL-1α (pg/cm^2^/min)^†^*	6.85 ± 0.13^a^ (940)	8.40 ± 0.14^b^ (4463)	8.82 ± 0.13^b^ (6739)	8.63 ± 0.15^b^ (5577)
IL-1β (pg/cm^2^/min)^†^*	− 4.09 ± 0.14^a^ (0.017)	− 5.04 ± 0.16^b^ (0.006)	− 4.70 ± 0.15^b^ (0.009)	− 5.97 ± 0.16^c^ (0.003)
IL-1ra (pg/cm^2^/min)^†*^	0.69 ± 0.19^a^ (2.00)	− 1.21 ± 0.20^b^ (0.30)	− 1.57 ± 0.19^b^ (0.21)	− 1.60 ± 0.22^b^ (0.20)
IL-6 (pg/cm^2^/min)*	0.0004 ± 0.00002^a^	0.0002 ± 0.00002^b^	0.0002 ± 0.00002^b^	0.0001 ± 0.00002^c^
IL-8 (pg/cm^2^/min)^†^*	− 4.36 ± 0.24^a^ (0.01)	− 8.69 ± 0.25^bc^ (0.0002)	− 7.92 ± 0.26^b^ (0.0004)	− 9.21 ± 0.31^c^ (0.0001)
IL-10 (pg/cm^2^/min)*	0.0014 ± 0.0002^a^	0.0006 ± 0.0001^b^	0.0006 ± 0.0001^b^	0.0003 ± 0.0002^b^

Values are LS means ± SE

^†^Data were analyzed on transformed scale and LS means provided on original scale in parenthesis. Box–Cox transformation technique was used to identify the optimal data transformation, where needed. ANOVA followed by Tukey’s HSD post hoc comparisons, where appropriate

*Significant main effects of site (*p* < 0.05)

^a,b,c^ LS means for sites sharing the same letter are not significantly different

### Sweat glucose

There was an effect of location on sweat glucose concentrations (*P* < 0.01) (Table 2) and excretion rates (*P* < 0.05) (Table 4). The FH had significantly lower glucose concentrations but higher excretion rates than all other locations. There were no differences among RDF, RS, and RT. There were no effects of time (Tables 3 and 5) or site*time on glucose concentration or excretion rates (*P* > 0.05).

**Table 5:** Sweat analyte excretion rates across time

	25 min	55 min	85 min
Cortisol (pg/cm^2^/min)^†^*	0.56 ± 0.14^a^ (1.76)	1.53 ± 0.10^b^ (4.61)	1.89 ± 0.10^c^ (6.63)
Glucose (ng/cm^2^/min)	6.88 ± 1.58^a^	8.84 ± 1.05^a^	9.24 ± 1.06^a^
EGF (pg/cm^2^/min)^†^*	− 1.96 ± 0.11^a^ (0.14)	− 2.61 ± 0.10^b^ (0.07)	− 3.36 ± 0.10^c^ (0.03)
IL-1α (pg/cm^2^/min)^†^	8.33 ± 0.12^a^ (4155)	8.24 ± 0.12^a^ (3777)	7.95 ± 0.12^a^ (2836)
IL-1ꞵ (pg/cm^2^/min)^†^	− 5.24 ± 0.14^a^ (0.005)	− 4.61 ± 0.13^a^ (0.010)	− 5.00 ± 0.13^a^ (0.007)
IL-1ra (pg/cm^2^/min)^†^	− 1.32 ± 0.18^a^ (0.27)	− 0.54 ± 0.17^a^ (0.59)	− 0.90 ± 0.17^a^ (0.41)
IL-6 (pg/cm^2^/min)	0.0002 ± 0.00002^a^	0.0003 ± 0.00002^a^	0.0002 ± 0.00002^a^
IL-8 (pg/cm^2^/min)^†^	− 8.02 ± 0.24^a^ (0.0003)	− 7.11 ± 0.23^a^ (0.0008)	− 7.50 ± 0.22^a^ (0.0006)
IL-10 (pg/cm^2^/min)	0.0006 ± 0.0001^a^	0.0009 ± 0.0001^a^	0.0008 ± 0.0001^a^

Values are LS means ± SE

^†^Data were analyzed on a transformed scale and LS means are provided on the original scale in parenthesis. Box–Cox transformation technique was used to identify the optimal data transformation, where needed. ANOVA followed by Tukey’s HSD post hoc comparisons, where appropriate

*Significant main effects of time (*p* < 0.05)

^a,b,c^ LS means for time points sharing the same letter are not significantly different

### Sweat cytokines

Because certain sweat cytokines may be undetectable if they fall outside of predetermined limits of sensitivity, we included cytokines for analysis where higher than 80% of samples returned values above the lower limit of detection (Supplemental Material: Table S1).

#### Concentration patterns

Sweat cytokine concentration patterns varied depending on the specific analyte and location. For most cytokines, including IL-1α (*P* < 0.0001), IL-1ꞵ (*P* < 0.0001), IL-1ra (*P* < 0.0001), IL-8 (*P* < 0.0001), and IL-10 (*P* < 0.05), concentrations were significantly impacted by site location (Table 2). Interestingly, the FH was significantly lower for IL-1α and IL-10 concentrations and significantly higher for IL-1ra and IL-8 concentrations compared to other sites. Sweat IL-ꞵ concentration was higher on the RS than the RT. EGF (*P* < 0.0001), IL-1ra (*P* < 0.0001), and IL-6 (*P* < 0.005) varied significantly over time (Table 3), where there was a flushing effect with concentrations significantly lower at 85 compared with 25. In addition, EGF concentrations were lower at 55 compared with 25. There were significant site*time interaction effects for IL-1ꞵ (*P* < 0.05) and IL-1ra (*P* < 0.05). IL-1ꞵ concentration was higher at the FH than the RT at time point 25. IL-1ra concentration was higher at the FH than all other sites at time point 25. Data across all sites and time points are shown in the Supplemental Material (Figures S1-10).

#### Excretion rate patterns

Excretion rates varied across sites for all cytokines: EGF (*P* < 0.0001), IL-1α (*P* < 0.0001), IL-1ꞵ (*P* < 0.0001), IL-1ra (*P* < 0.05), IL-6 (*P* < 0.0001), IL-8 (*P* < 0.0001), and IL-10 (*P* < 0.05) (Table 4). For all cytokines except IL-1α, excretion rates were higher on the FH than all other all sites. IL-1α excretion rate was lower on the FH than all other sites. In addition, IL-1ꞵ and IL-6 excretion rates were lower on the RT than the RDF and RS. IL-8 excretion rates were lower on the RT than the RS. EGF was the only detectable cytokine to demonstrate differences in excretion rate over the exercise duration (*P* < 0.0001) (Table 5). There were significant site*time interaction effects for IL-1α (*P* < 0.05) and IL-1ꞵ (*P* < 0.05). IL-1α excretion rate was lower on the FH than the RDF and RS at time point 55. IL-1α excretion rate was lower on the FH than the RS at time point 85. At time point 25, IL-1ꞵ excretion rate was lower on the RT than the FH and RS, and lower on the RDF than the FH. IL-1ꞵ excretion rate was lower on the RT than all other sites at time point 85.

## Discussion

There is considerable interest in the potential for sweat to serve as a non-invasive biofluid, yet the time course changes and regional differences in sweat biomarkers have been largely uninvestigated. This is the first study to include the concentration and excretion rates of sweat cortisol, glucose, and select cytokines at four anatomical locations over three distinct time points during exercise. Contrary to our original hypothesis, the time course of sweat sampling played a relatively small role in sweat cytokine concentration and excretion rates, with the exception of EGF. Further, sweat cortisol was the sole analyte to increase as exercise duration progressed, while glucose concentration and excretion rates remained similar over time. In support of previous research, we demonstrated the importance of sweat sample location, as evidenced by the unique differences at the FH which were present across most sweat analytes. Overall, we demonstrated that sweat sample concentration and excretion rate patterns are highly dependent on the specific sweat analyte and may be influenced by the selected sample location and time point. This is essential information for future work that plans to utilize sweat as a diagnostic biomarker.

The first novel finding of this study was that the time course of sweat sampling had a lesser effect than anatomical location on measured cytokine concentrations. We originally hypothesized that cytokine values, particularly IL-1α (Sato and Sato 1994), may demonstrate a “flushing effect” where concentrations appear higher in earlier sweat samples due to their presence in the stratum corneum or residual sweat in the ductal lumen (Baker 2019). However, EGF, IL-1ra, and IL-6 were the only cytokines to display this pattern. The most pronounced flushing effect was demonstrated with EGF, where mean values at 85 min were only 16% of the initial sample collected at 25 min of exercise (Table 3).

EGF has an important role in keratinocyte proliferation, differentiation, and wound healing, and is vital to the development of sweat gland buds or cells (Li et al. 2002; Matsumura et al. 2017; Shikiji et al. 2003). It may also relieve inflammation in skin conditions like atopic dermatitis (Kim et al. 2018). The role of EGF in exercise is unclear, but it is likely that the type of exercise stimulus dictates the response (Diaz-Castro et al. 2020; Yasar et al. 2021). The decrease in sweat EGF concentration over time in the present study may represent a flushing effect of the sweat duct (Baker and Wolfe 2020). The effect of sweat gland flushing has been previously identified in multiple sweat analytes (Boysen et al. 1984; Ely et al. 2011; Paulev et al. 1983). Higher concentrations early in sweat sampling may also be present due to methodological considerations. It is unlikely the pattern of appearance described here is due to surface contamination, because a skin cleaning protocol was followed prior to the placement of each sweat patch (as described in (Baker et al. 2018)), although surface contamination cannot be ruled out altogether.

Because of their large molecular mass, passage of cytokines from the interstitial fluid to the lumen of the eccrine secretory coil is difficult. Instead, cytokines and chemokines are thought to originate from the eccrine gland itself, since certain proteins can be produced there (Aranyosi et al. 2021; Jones et al. 1995; Sato and Sato 1994). Thus, it seems likely that EGF concentrations described here are derived from the eccrine gland (Saga and Jimbow 2001; Saga and Takahashi 1992), thus influencing the sweat content and pattern of appearance (Baker and Wolfe 2020). If this pattern is derived from the eccrine gland, EGF concentration and excretion rates may reflect suppression via cortisol or other cytokines, since values declined as exercise time continued.

In addition to EGF, other cytokines measured in the current study may be particularly important in monitoring skin conditions (Aranyosi et al. 2021; Dai et al. 2013). This is especially true of the IL-1 family of cytokines, where dysregulation may lead to a variety of skin conditions, such as psoriasis, atopic dermatitis, and cutaneous lupus erythematosus (Cai et al. 2019; Jensen 2010). It has been suggested that the cytokines in the sweat may originate from contracting muscle, enter the systemic circulation, and get secreted into the eccrine gland to form primary sweat. However, it seems more likely that cytokines present in the sweat are resident in the skin and function as surveillance molecules to signal local wound injury or repair (Dai et al. 2013). Previous evidence suggests that exercise or heat stress induces local production of IL-1 in the eccrine glands (Didierjean et al. 1990). Our results build on this finding and demonstrate that this response is specific to the sweat sample location and the particular cytokine in the IL-1 family.

Certain cytokines, like IL-6 and IL-1ra, are known to increase in the circulation with exercise intensity and duration (Pedersen et al. 2001). In our sweat samples, these interleukins decreased over time, suggesting that content in sweat samples may not reflect blood concentrations. Alternatively, the cytokine response in sweat could reflect local or systemic suppression. Cortisol may be responsible for this suppression, as it is activated by IL-6 and negatively feeds back to suppress further cytokine expression (Bethin et al. 2000; Steensberg et al. 2003; Stouthard et al. 1995). Cortisol is a known cytokine modulator and has been shown to promote IL-1ra, IL-4, and IL-10 and inhibit TNF-α, IL-1β, IFN-α, IFN-γ, and IL-2 (Bethin et al. 2000; Suzuki et al. 2002). These actions of cortisol have the potential to be reflected in primary sweat.

The research describing the concentration of cytokines in sweat is fairly limited. Two studies that examined resting cytokine sweat concentrations in healthy premenopausal females over a 24 h period, placed sweat patches on the rib cage above the waist and found similar IL-1β values to those reported here (Cizza et al. 2008; Marques-Deak et al. 2006). While other cytokine values reported including TNF-α, IL-8, and IL-6 were considerably higher, IL-1α was orders of magnitude lower than our present results (Marques-Deak et al. 2006). Another study that examined IL-1 values in eccrine sweat following active and passive modes of sweat induction found comparable values to our results of both IL-1α and IL-1β utilizing the pouch technique with oil for sweat collection from sites on the torso (Didierjean et al. 1990). Discrepancies in reported sweat cytokine concentration could be explained by differences in exercising versus resting conditions (Sato et al. 1989), the duration of sweat patch adherence and location on the body (Baker 2019; Barnes et al. 2016), and methodological analysis of cytokines (recycling immunoaffinity chromatography, enzyme immunoassay, or multiplex). Variability in cytokine measures may also be reflective of sex, age, or differences in fitness levels.

Although not directly tested here, if sweat cytokine concentrations are reflective of concentrations in the blood (Cizza et al. 2008), our results may reflect these circulating patterns. TNF-α and IL-1β expression in the skeletal muscle increases following exercise, although most exercise studies show that circulating concentrations of these cytokines, along with IFN-γ, are relatively small or unchanged over an exercise bout (Pedersen and Toft 2000; Suzuki et al. 2002). Our results are in agreement with these exercise studies as TNF-α and IFN-γ were not detectable in sweat samples (Supplemental Material: Table S1). Conversely, IL-1β was detected in sweat samples, albeit at lower concentrations than previously reported (Dai et al. 2013; Didierjean et al. 1990).

Our next major finding demonstrated that cortisol concentration and excretion rates were affected by time. Relationships between cortisol concentrations in blood plasma and sweat cortisol have been previously examined (Torrente-Rodriguez et al. 2020), but standard ranges for cortisol obtained from sweat samples throughout the course of exercise at multiple locations have not been determined. Like cytokines, the passage of cortisol from the interstitial fluid to the lumen of the secretory coil of the eccrine gland is difficult. This is owing to cortisol’s large molecular mass and because it is mostly protein-bound (Jenkins et al. 1969). Instead, sweat cortisol may originate from the skin or eccrine sweat gland itself (Hirasawa et al. 1997). Cortisol concentrations reported here are generally congruent with other reported values following exercise induced sweating (Pearlmutter et al. 2020; Torrente-Rodriguez et al. 2020) as well as passive heating (Jia et al. 2016), despite cortisol quantification being conducted via a variety of methods (Jia et al. 2016; Pearlmutter et al. 2020; Torrente-Rodriguez et al. 2020). While these studies are relatively similar in cortisol concentration, the timing of sample collection, method of sweat induction, and sampling techniques varied.

The impact of different methodologies heavily influences sample concentration and are important to discuss (Baker and Wolfe 2020). When sweat samples were collected by rubbing a cotton swab over the scalp hair and neck (until saturation) immediately after ~ 10–60 min of vigorous exercise, cortisol values varied drastically (~ 8–142 ng/ml (Russell et al. 2014)) in comparison to those reported here. A similar sweat sampling technique where a cotton swab was gently moved over the FH and neck following a 30 min run (at 65% VO_2 max_) also yielded high sweat cortisol concentrations (Russell et al. 2014). Although circadian rhythms may have influenced these results, since examination times varied throughout the day (8:45 a.m.-7:45 p.m.). The results of previous studies may also be influenced by sample contamination caused by the presence of cortisol in or by keratinocytes (Terao and Katayama 2016). Alternatively, sweat cortisol concentrations reported in the present study may actually be blunted by individual training status, since cortisol activation is inversely proportional to the level of physical training (Luger et al. 1987).

Intake of a carbohydrate solution during exercise may have also blunted cortisol concentration and the cytokine response reported here. Maintenance of blood glucose levels during exercise can attenuate increases in circulating cortisol and the initiation of the cytokine stress response (Nehlsen-Cannarella et al. 1997), potentially decreasing subsequent appearance in sweat. Relative to blood, concentrations of glucose in sweat are known to be very low (Boysen et al. 1984). However, unlike cortisol and cytokines, the primary source of sweat glucose is likely the blood, as sweat glucose does not seem to be influenced by epidermal contamination or the eccrine sweat gland itself (Baker and Wolfe 2020). Sweat glucose concentrations reported here are within ranges previously described (Baker and Wolfe 2020), but were not altered by sample collection time.

A key finding that is consistent across this study highlights the unique sweat profile of the FH. Although high LSRs at the FH have been previously demonstrated (Baker et al. 2018; Kondo et al. 1998), the underlying mechanisms are not well described. LSRs are the result of the density of active sweat glands and the secretion rate per gland (Baker and Wolfe 2020; Kondo et al. 1998). The density of sweat glands is higher on the FH compared to other sites and maximal LSR appears to be higher on the FH than on the forearm and back (Sato and Dobson 1970). This implies that the high density of activated FH sweat glands may have largely influenced these results. Importantly, higher density of sweat glands does not necessarily translate to a higher sweating rate, and in fact, differences in LSRs may be due to sweat output per gland instead of the number of total active glands (Baker 2019). LSR patterns and values differed across time and location as expected. These patterns are in accordance with previous findings where the FH displayed the highest LSR followed by the RDF, RS, and RT (Baker et al. 2018). High FH LSRs may also be influenced by the local skin temperature and mental or emotional stressors (Kondo et al. 1998). It is unknown why the FH is so densely populated with sweat glands, but it has been hypothesized that increased FH sweating may be an evolutionary mechanism utilized to keep the brain cool (Cabanac et al. 1987).

The FH is also unique in its composition, as the regional differences in concentrations found for some analytes were largely driven by the FH site. Relative to other sites, the FH is rich in sebaceous and apocrine glands, which secrete a viscous protein- and lipid-rich fluid that could elevate the total sample volume or concentration of certain analytes (Baker and Wolfe 2020; Saga and Takahashi 1992). The high LSR of the FH could also have a direct or indirect impact on sweat analyte concentrations. As discussed, cortisol and cytokine concentrations are derived at least in part from the sweat gland itself. Thus, it is conceivable that greater sweat gland activity may result in higher production and therefore higher concentrations of analytes, as was observed with cortisol and some cytokines (IL-1ra and IL-8) on the FH in the present study. It is unclear why the concentrations of other cytokines (IL-1α and IL-10) were lowest on the FH but could be explained by a dilution effect from the large volume of sweat output on the FH. Likewise, sweat glucose concentrations on the FH may have been diluted by the high sweat volume. It is of note, however, that despite slightly lower sweat glucose concentrations on the FH, glucose excretion rate was significantly higher at the FH than other sites, owing to the higher LSR on the FH. Future research should use caution and consider the potential confounding factors associated with the FH (e.g., contamination from non-eccrine gland secretions and impact of high LSR) if using this site for diagnostic sweat sampling.

## Considerations and future directions

This study raises some important considerations. Our sample size did not allow for comparisons between men and women, although this should be examined in future studies as certain sweat analytes may be affected by sex differences (Benini et al. 2015). Further, some sweat analytes, such as cortisol, are influenced by circadian rhythms (Jia et al. 2016). In the current study, six out of eight subjects underwent testing at ~ 0900 h; however, the other two subjects underwent testing at ~ 1300 h. Although variability in cortisol concentrations was not as high as those previously reported when morning and night testing were included (Grass et al. 2015; Russell et al. 2014), we cannot rule out this as a potential source of variation. It is important to note that although these differences may impact absolute values, they are unlikely to affect the within subject comparisons made here.

## Conclusion

This study described the influence of sweat sampling time points and locations on the concentrations and excretion rates of cortisol, glucose, and select cytokines. Here, we demonstrate the importance of sweat sample location as evidenced by the unique sweat profile and high LSR of the FH. Sweat cortisol, IL-1ra, and IL-8 concentrations were higher on the FH, while sweat glucose, IL-1α, and IL-10 concentrations were lower on the FH than most other sites. However, owing to the FH’s drastically higher LSR, overall excretion rates were significantly higher than other sites for almost all analytes (with the exception of IL-1α). Sweat cortisol increased over time, whereas the cytokines EGF, IL-1ra, and IL-6 decreased throughout the course of exercise. This study is critical for future work in this area, as it reports not only an array of sweat cytokine concentrations at specific times and locations, but also describes sweat analytes such as cortisol and glucose that are known to modulate cytokine responses. Describing the time course and location of sweat constituents is a vital step in understanding the utility of sweat as a useful diagnostic tool.

## Supplementary Information

Below is the link to the electronic supplementary material.

Supplementary file1 (DOCX 28 KB)

Supplementary file2 (DOCX 208 KB)

## Data Availability

All data generated or analyzed during this study are included in this published article and supplementary files.

## Abbreviations

ANOVA: Analysis of variance
CV: Coefficient of variation
EGF: Epidermal growth factor
ELISA: Enzyme-linked immunoassay
FH: Forehead
HR: Heart rate
IFN: Interferon
IL: Interleukin
LSR: Local sweating rate
ra: Receptor agonist
RDF: Right dorsal forearm
RPE: Rating of perceived exertion
RS: Right scapula
RT: Right triceps
SE: Standard error
TNF: Tumor necrosis factor
USG: Urine-specific gravity
VO2max: Maximal oxygen uptake
