# Supplementary material for: Regional and time course differences in sweat cortisol, glucose, and select cytokine concentrations during exercise
Source: Eur J Appl Physiol. 2023 Apr 2;123(8):1727–38. doi: 10.1007/s00421-023-05187-3 (PMC10363073; doi:10.1007/s00421-023-05187-3)
Supplement: Supplementary file 1 — Supplementary file1 (DOCX 28 KB) [file 421_2023_5187_MOESM1_ESM.docx]

**Supplemental Information (Table S1)**

Regional and Time Course Differences in Sweat Cortisol, Glucose, and Select Cytokine Concentrations during Exercise

Michelle A. King^1^, Shyretha D. Brown^1^, Kelly A. Barnes^1^, Peter John D. De Chavez^2^, Lindsay B. Baker^1^

^1^Gatorade Sports Science Institute, PepsiCo R&D Life Sciences, Barrington, IL, USA

^2^Data Science and Analytics, PepsiCo R&D, Barrington, IL, USA

Address for correspondence:

Lindsay Baker

50 E Stevens Ave

Valhalla, NY 10595

Email: lindsay.baker@pepsico.com

| **Table S1.** Cytokine markers considered for inclusion with median concentrations | | | |  |
| --- | --- | --- | --- | --- |
|  | Lower limit of detection (pg/ml) | Accepted?  (% returned) | Sweat concentration (pg/ml)  (Median ± IQR) | |
| **EGF** | 1.07 | Yes (99%) | 42.0 ± 65.2 | |
| **IFNγ** | 2.22/0.13 | No (48%) | ND | |
| **IL-1α** | 0.69 | Yes (98%) | 3413 ± 4564 | |
| **IL-1β** | 0.02/1.43 | Yes (96%) | 5.1 ± 9.1 | |
| **IL-1ra** | 0.4 | Yes (98%) | 343.4 ± 717.2 | |
| **IL-6** | 0.04 | Yes (100%) | 0.12 ± 0.03 | |
| **IL-8** | 0.09/0.01 | Yes (93%) | 0.21 ± 2.07 | |
| **IL-10** | 0.02/0.01 | Yes (83%) | 0.18 ± 0.7 | |
| **TNFα** | 0.01/3.75/1.64 | No (52%) | ND | |
