# Supplementary material for: Regional and time course differences in sweat cortisol, glucose, and select cytokine concentrations during exercise
Source: Eur J Appl Physiol. 2023 Apr 2;123(8):1727–38. doi: 10.1007/s00421-023-05187-3 (PMC10363073; doi:10.1007/s00421-023-05187-3)

**Supplemental Information (Figures S1-10)**

Regional and Time Course Differences in Sweat Cortisol, Glucose, and Select Cytokine Concentrations during Exercise

Michelle A. King^1^, Shyretha D. Brown^1^, Kelly A. Barnes^1^, Peter John D. De Chavez^2^, Lindsay B. Baker^1^

^1^Gatorade Sports Science Institute, PepsiCo R&D Life Sciences, Barrington, IL, USA

^2^Data Science and Analytics, PepsiCo R&D, Barrington, IL, USA

Address for correspondence:

Lindsay Baker

50 E Stevens Ave

Valhalla, NY 10595

Email: lindsay.baker@pepsico.com


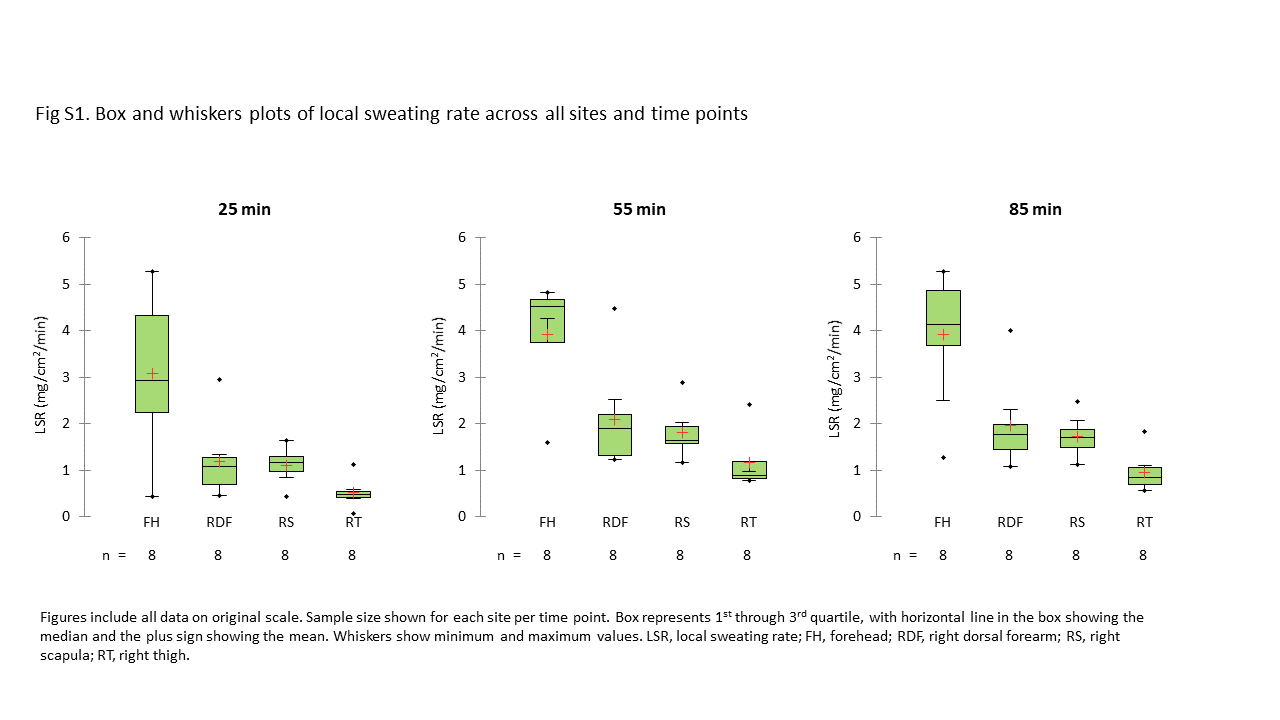


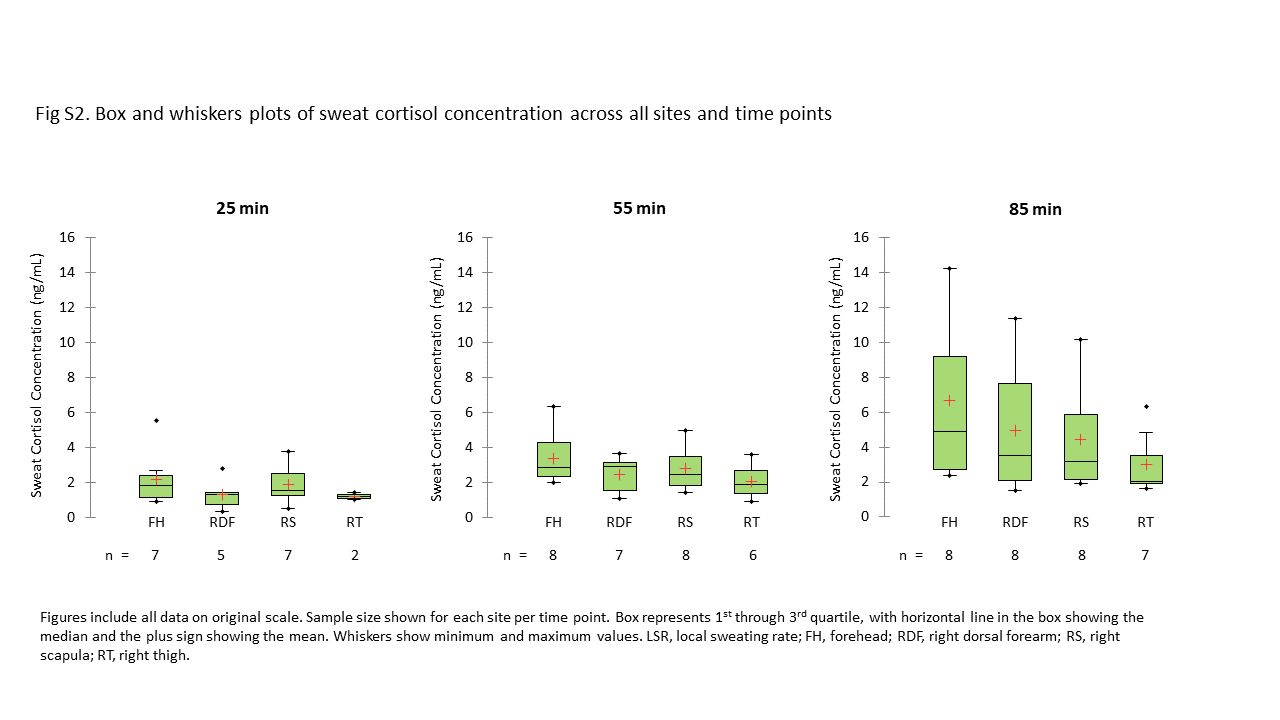


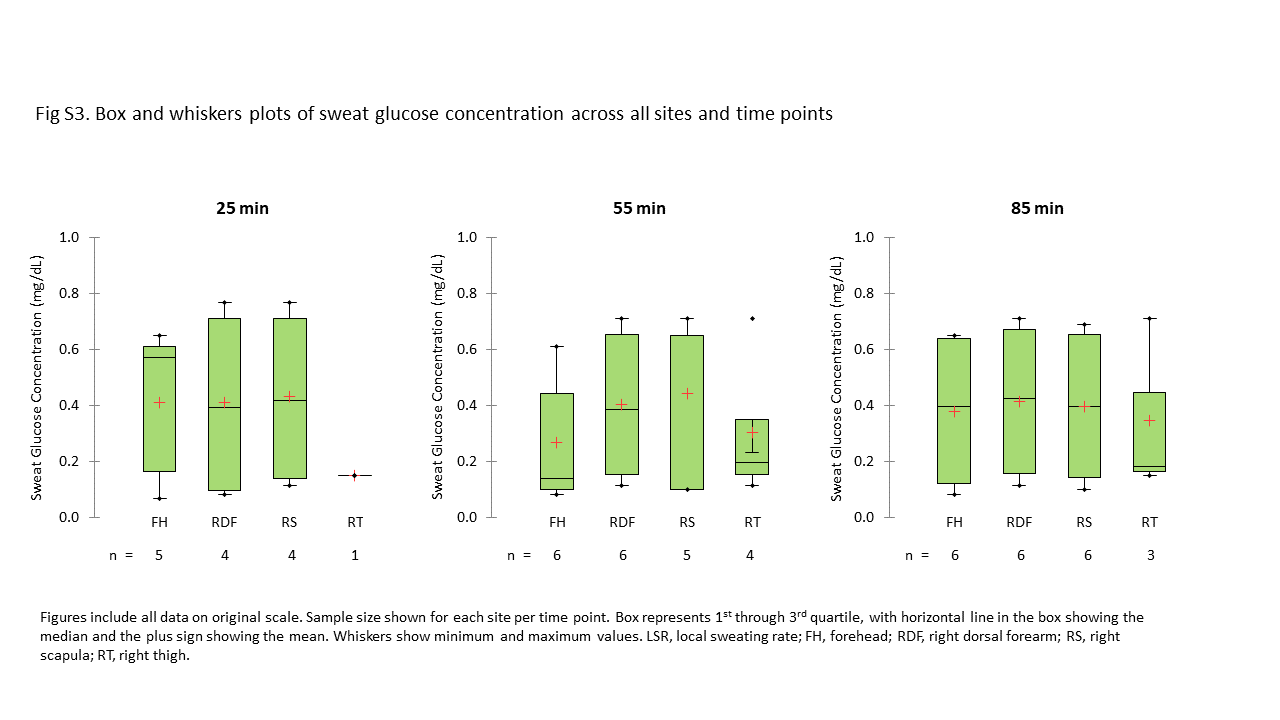


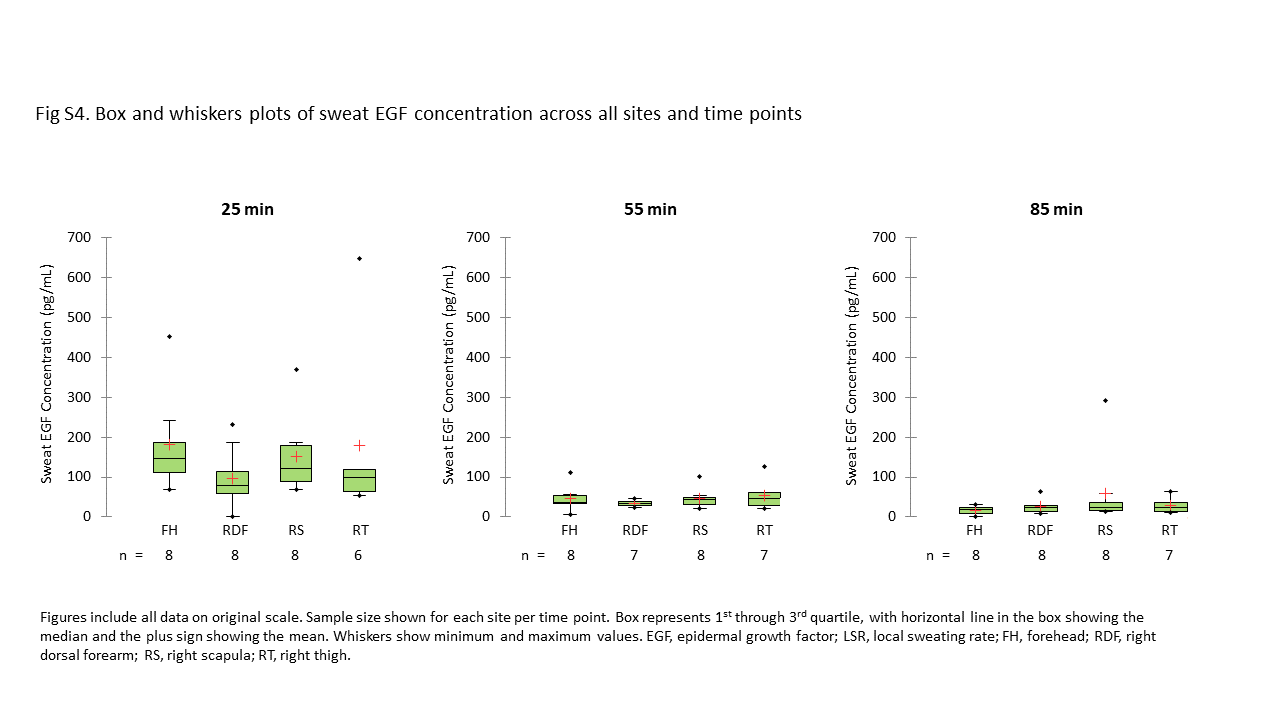


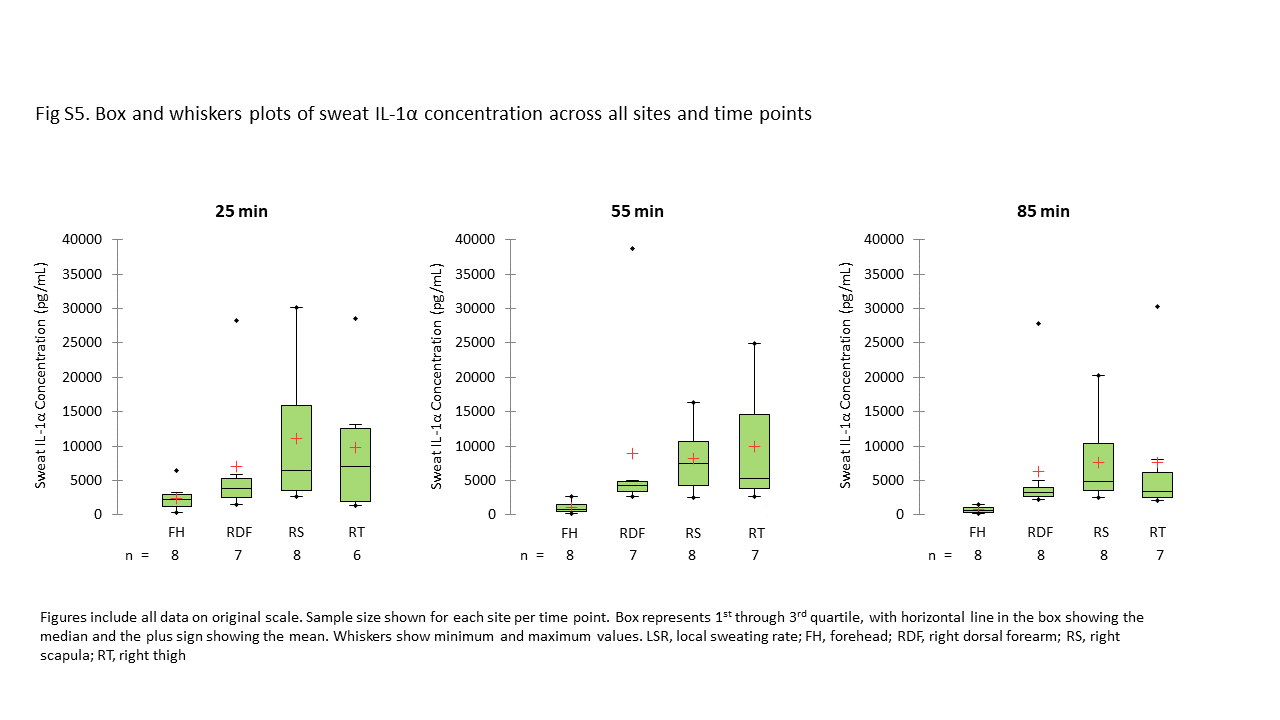


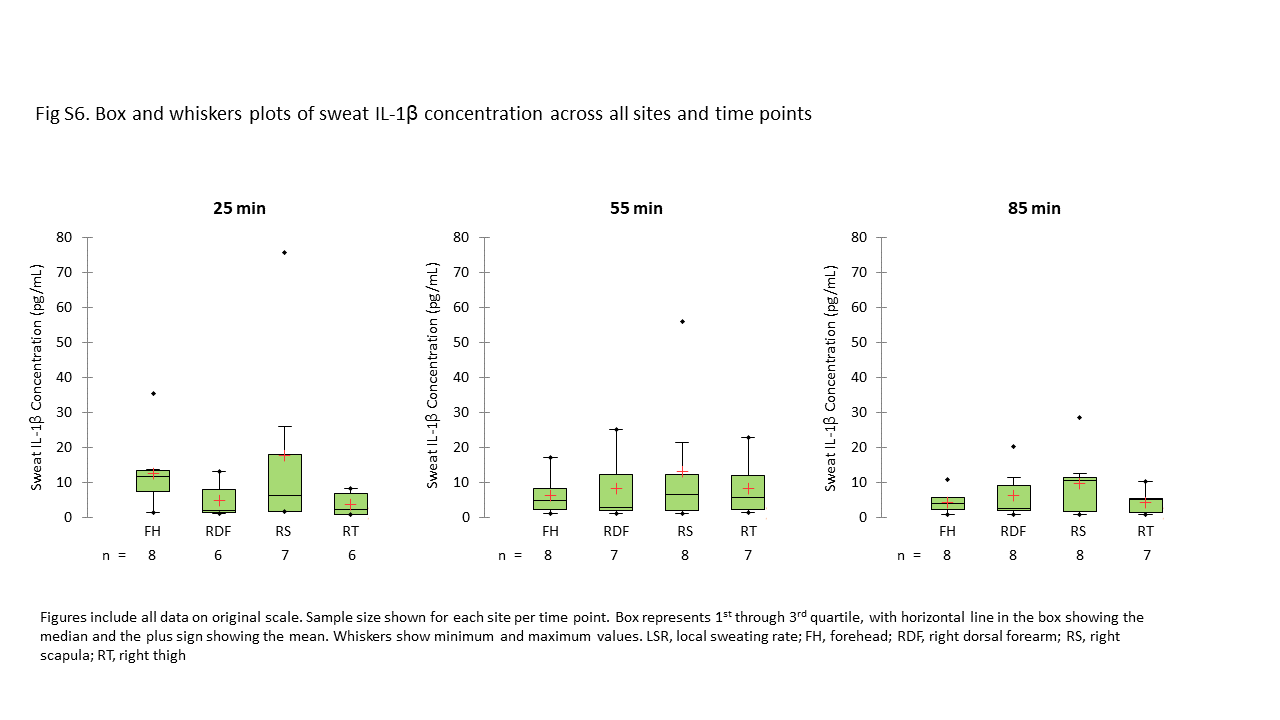


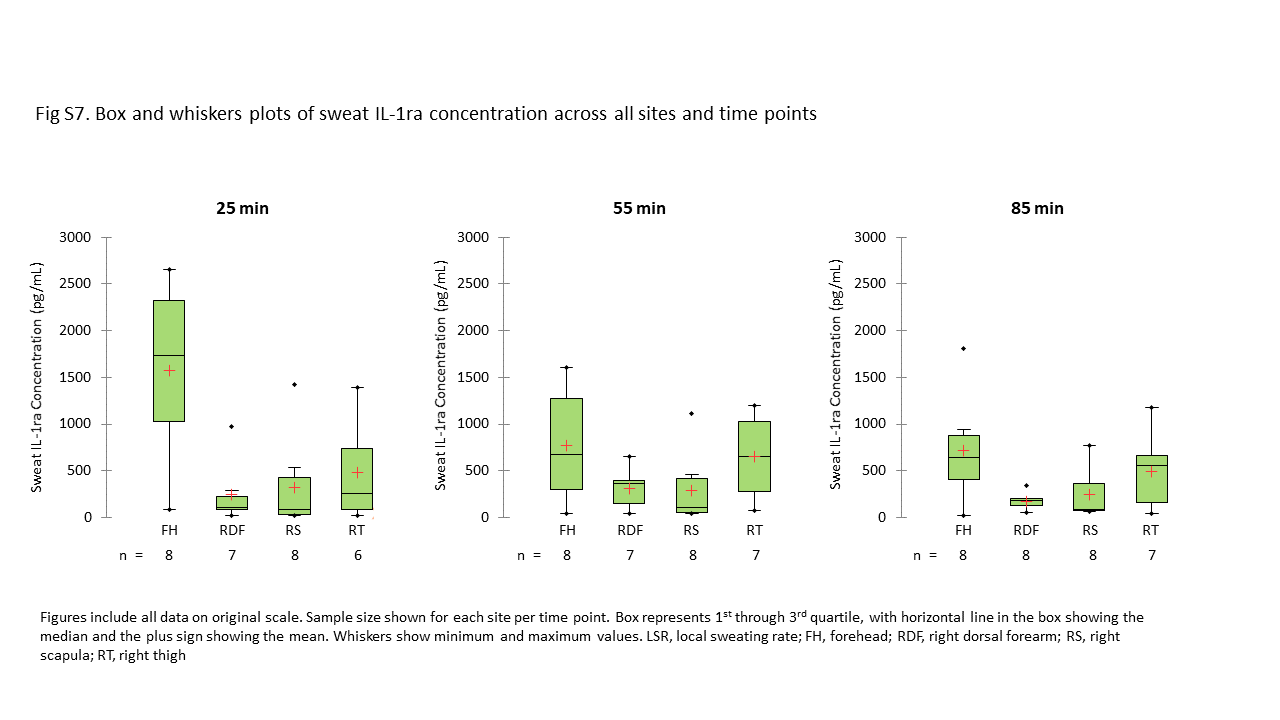


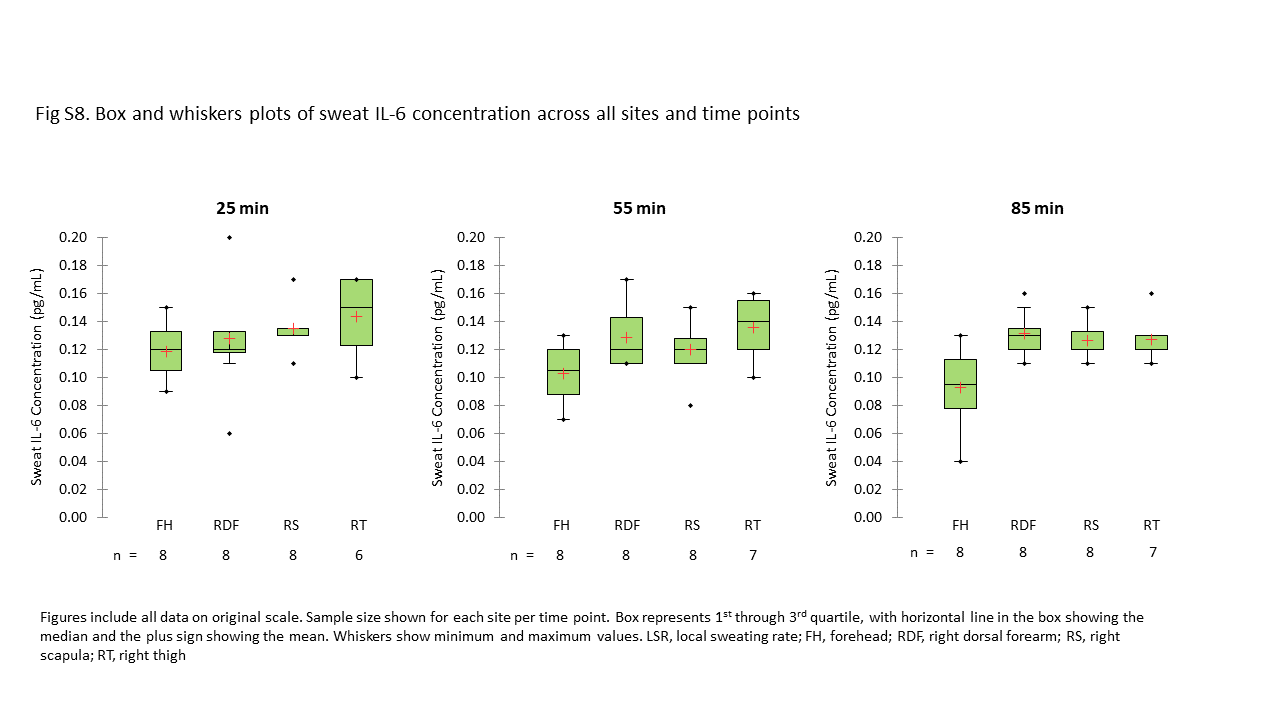


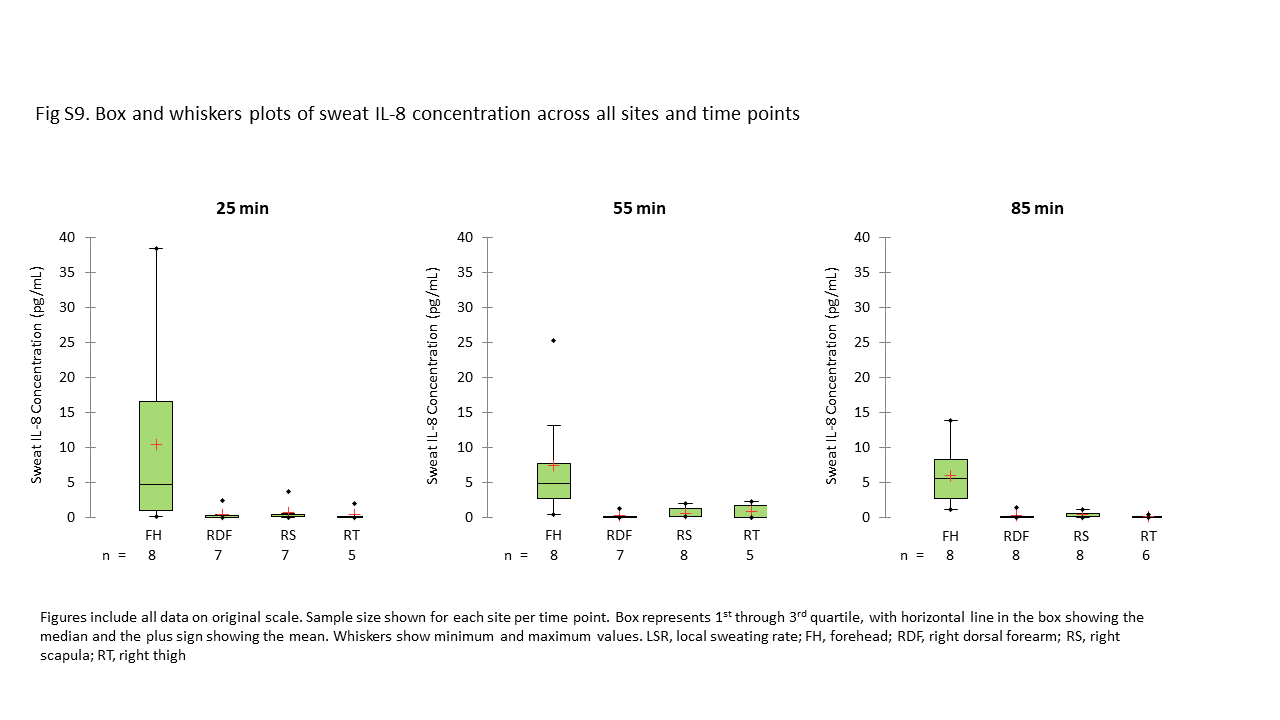


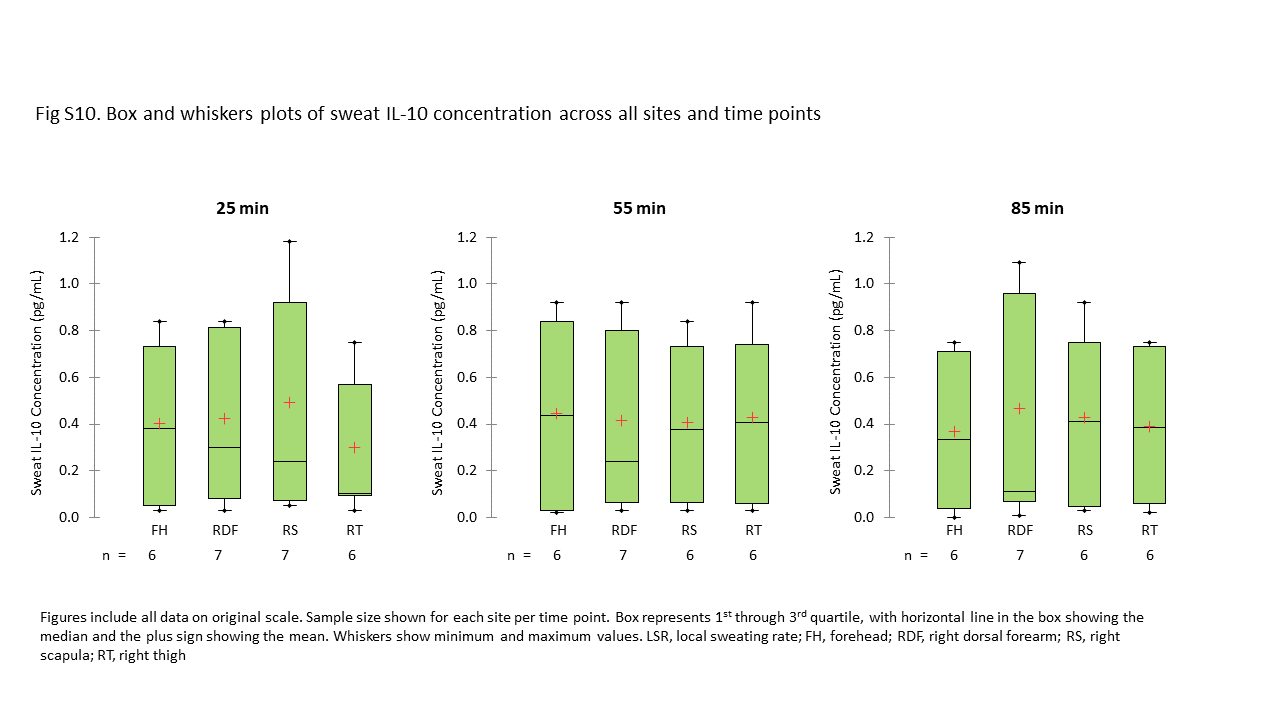

Supplement: Supplementary file 2 — Supplementary file2 (DOCX 208 KB) [file 421_2023_5187_MOESM2_ESM.docx]
